# 3-Hydroxypropionic Acid Enhances Hair Growth-Related Signaling in Human Follicle Dermal Papilla Cells via Activation of the Wnt/β-Catenin Pathway

**DOI:** 10.3390/ijms27031480

**Published:** 2026-02-02

**Authors:** Chae Young Jeon, Yun Hoo Jo, Seung A. Woo, Yura Lee, Woochul Jung, Dong Wook Shin

**Affiliations:** 1 Research Institute for Biomedical and Health Science, Konkuk University, Chungju 27478, Republic of Korea; young4mam@kku.ac.kr (C.Y.J.); julie3734@kku.ac.kr (Y.H.J.); 2 Advanced Materials R&D Center, LG Chem, 30, Magokjungang 10-ro, Gangseo-gu, Seoul 07796, Republic of Korea; wooseunga@lgchem.com (S.A.W.); yuralee@lgchem.com (Y.L.); woochuljung@lgchem.com (W.J.)

**Keywords:** 3-hydroxypropionic acid, oxidative stress, human follicle dermal papilla cells, hair growth, Wnt/β-catenin signaling

## Abstract

Hair loss is a common condition that affects a large number of people worldwide, impacting both men and women. Its development is closely linked to the function of hair follicle dermal papilla cells (HFDPCs), which play a pivotal role in maintaining hair growth and follicle integrity. However, these cells are particularly vulnerable to oxidative stress generated under psychological or environmental stressful conditions. Preserving the mitochondrial function and biological activity of HFDPCs is critical for preventing stress-related hair loss. This study investigated the protective and hair growth-promoting effects of 3-hydroxypropionic acid (3-HP), a naturally occurring organic acid with antioxidant potential, on HFDPCs exposed to H_2_O_2_-induced oxidative stress conditions. Treatment with 3-HP significantly enhanced cell viability and migration in H_2_O_2_-damaged HFDPCs. In addition, 3-HP reduced intracellular reactive oxygen species (ROS) accumulation and improved mitochondrial membrane potential as well as ATP production. Furthermore, 3-HP upregulated alkaline phosphatase (ALP) expression and activated hair growth-related signaling pathways, including the Wnt/β-catenin axis. Finally, treatment with 3-HP resulted in a significant enlargement of three-dimensional spheroids in H_2_O_2_-damaged HFDPCs. These findings suggest that 3-HP mitigates oxidative stress-induced damage and promotes hair follicle cell function, indicating its promise as a treatment option for improving oxidative stress-related hair loss conditions.

## 1. Introduction

Hair loss (alopecia) is highly prevalent and adversely affects psychological well-being and quality of life. Hair follicles undergo distinct growth, regression, and resting phases—anagen, catagen, and telogen-during which the dermal papilla functions as a central mesenchymal niche coordinating epithelial-mesenchymal interactions [1,2]. HFDPCs reside in the dermal papilla and contribute to the induction and sustainment of the anagen phase through the secretion of diverse growth factors and extracellular matrix components that regulate neighboring epithelial cells [3,4]. They secrete a wide range of growth factors, including vascular endothelial growth factor (VEGF) and insulin-like growth factor-1 (IGF-1), which are crucial for hair follicle development and hair shaft elongation [5,6]. The functionality of HFDPCs is influenced by various intrinsic and extrinsic factors, with oxidative stress recognized as a major contributor [7]. Oxidative stress reflects a state in which reactive oxygen species (ROS) overwhelm endogenous antioxidant systems [8]. Excessive ROS can damage cellular components, including lipids, proteins, and DNA, leading to impaired cell function or apoptosis [9]. Such oxidative injury has been closely associated with hair follicle miniaturization, premature entry into catagen, and the progression of alopecia [10]. Previous reports indicate that HFDPCs derived from balding scalp tissue are more vulnerable to oxidative stress, leading to enhanced secretion of transforming growth factor-beta (TGF-β), a well-established inhibitor of hair growth, and accelerated cellular senescence [11,12]. Furthermore, mitochondrial dysfunction in HFDPCs can exacerbate ROS production, creating a vicious cycle that impairs hair follicle regeneration [13,14]. Activation of the nuclear factor erythroid 2-related factor 2 (Nrf2) signaling pathway enhances cellular resistance to oxidative damage through the induction of antioxidant and cytoprotective enzymes [15,16]. Activation of Nrf2 signaling in HFDPCs is therefore considered a key protective response that not only restores redox homeostasis but also helps preserve their inductive potential, thereby supporting hair growth and follicular health [17,18].

Recent studies show that oxidative stress not only disrupts intracellular redox balance but also impairs growth-related signaling networks that are essential for follicular maintenance [19]. Among these networks, the Wnt/β-catenin pathway plays a pivotal role in initiating anagen, sustaining dermal papilla inductivity, and supporting epithelial cell proliferation [20]. Reduced Wnt/β-catenin activity has been observed in prematurely regressing or miniaturized follicles, indicating that oxidative stress can suppress canonical Wnt signaling and thereby hinder transcriptional programs required for hair growth [21]. Growing evidence also demonstrates functional crosstalk between Nrf2-mediated antioxidant defenses and Wnt/β-catenin signaling [22,23]. Nrf2 activation can stabilize β-catenin and enhance the expression of its downstream targets under oxidative conditions, whereas diminished Nrf2 activity increases susceptibility to ROS-induced β-catenin degradation [24,25]. This reciprocal interaction suggests that maintaining redox homeostasis is critical for preserving the Wnt-dependent regenerative capacity of dermal papilla cells [26].

Given the detrimental effects of oxidative stress on HFDPCs and hair follicle integrity, there is increasing interest in identifying compounds capable of mitigating ROS-induced cellular damage [27]. Natural antioxidants have been widely investigated for their potential to protect HFDPCs from oxidative stress and thereby preserve hair follicle function [28,29]. 3-Hydroxypropionic acid (3-HP) has recently attracted attention as a bioactive molecule with putative antioxidant properties [30]. Although some biochemical studies have noted that 3-HP participates in cellular metabolic and redox-related pathways [31,32], these findings do not establish a defined antioxidant mechanism and have not been evaluated in hair follicle systems. Furthermore, unlike conventional thiol-based antioxidants such as glutathione or N-acetylcysteine, the mechanistic basis of 3-HP’s antioxidant actions remains unclear, and any potential distinctions should be considered hypothetical at this stage.

In this study, we aimed to elucidate the protective and regenerative effects of 3-HP on HFDPCs subjected to oxidative stress induced by hydrogen peroxide (H_2_O_2_). We hypothesized that 3-HP could mitigate oxidative damage, enhance mitochondrial function, and promote the expression of hair growth-related markers in HFDPCs. Insights into the function of 3-HP may contribute to the advancement of alternative approaches for managing oxidative stress-induced hair loss.

## 2. Results

### 2.1. The Cell Viability of HDPCs Treated by 3-HP

**Figure 1:**
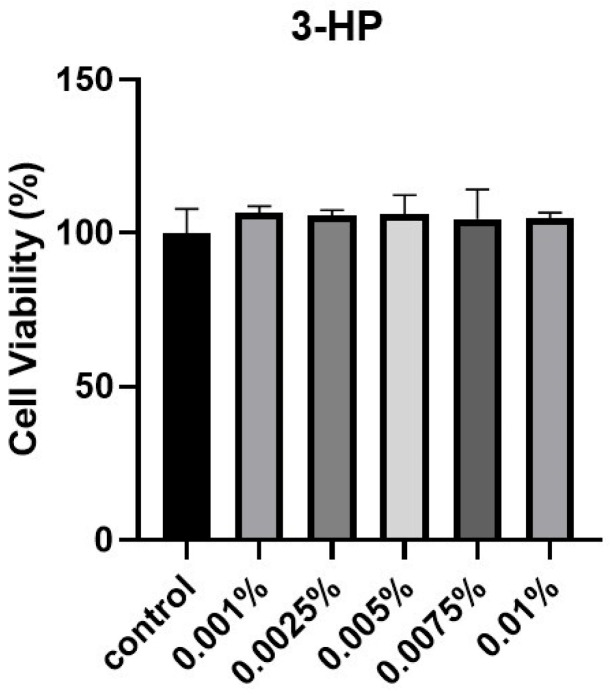
The influence of 3-HP on cellular viability. HFDPC viability was assessed via the EZ-Cytox assay following treatment with various concentrations of 3-HP for 24 h. This experiment was performed with at least three (*n* ≥ 3).

To evaluate the effect of 3-HP on cell viability, HFDPCs were subjected to the EZ-Cytox cell viability assay. Cells treated with 3-HP exhibited higher viability compared with the untreated control group (Figure 1). Based on these results, concentrations from 0.005 to 0.01% were selected for subsequent experiments. In the preliminary cytotoxicity Test, concentrations above 0.02% significantly reduced cell viability, indicating potential cytotoxic effects. Therefore, 0.005–0.01% were selected as non-toxic and physiologically relevant concentrations for subsequent experiments.

### 2.2. 3-HP Enhanced the Cell Wound Healing Ability of HFDPCs Damaged by H_2_O_2_

**Figure 2:**
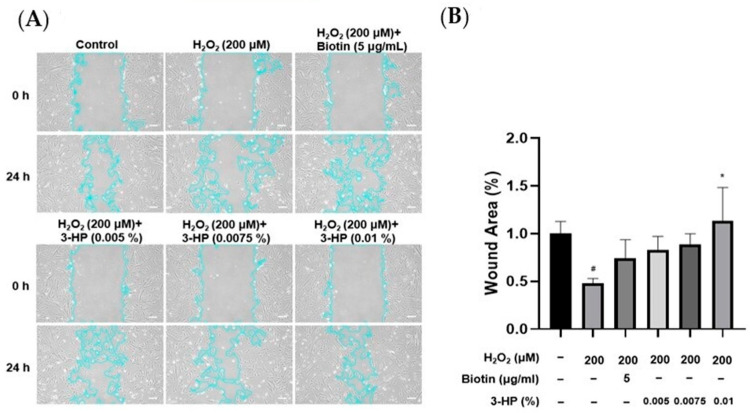
The effect of 3-HP on enhancing cell migration in H_2_O_2_-damaged HFDPCs. (**A**) Cell migration was assessed at 0 and 24 h in HFDPCs exposed to oxidative stress induced by H_2_O_2_ and subsequently treated with 3-HP at concentrations ranging from 0.005% to 0.01%. The outlines of the cells are highlighted in light blue. (**B**) Quantitative analysis of the wound area (%), calculated using ImageJ software (version 1.53e). Data were presented as the mean ± SD (n = 3). * *p* < 0.05 vs. H_2_O_2_-treated group, # *p* < 0.05 vs. control group. Scale bar: 20 µm.

The migratory capacity of HFDPCs is essential for hair growth, as increased mobility of these cells is closely linked to the expansion of dermal papilla volume, which plays a pivotal role in regulating follicle size and hair shaft formation [33,34]. To evaluate the effect of 3-HP on wound healing, the cell migration of HFDPCs was evaluated under oxidative stress triggered by H_2_O_2_. A linear scratch was created on a confluent cell monolayer using a sterile pipette tip, and wound closure was monitored over 24 h. Compared with cells treated with H_2_O_2_ alone, the 3-HP-treated group demonstrated a significantly higher rate of wound closure, indicating enhanced migratory activity (Figure 2). These results indicate that 3-HP enhances the migratory capacity of HFDPCs and facilitates gap closure in an oxidative stress environment.

### 2.3. 3-HP Promoted ALP Expression in H_2_O_2_-Damaged HFDPCs

**Figure 3:**
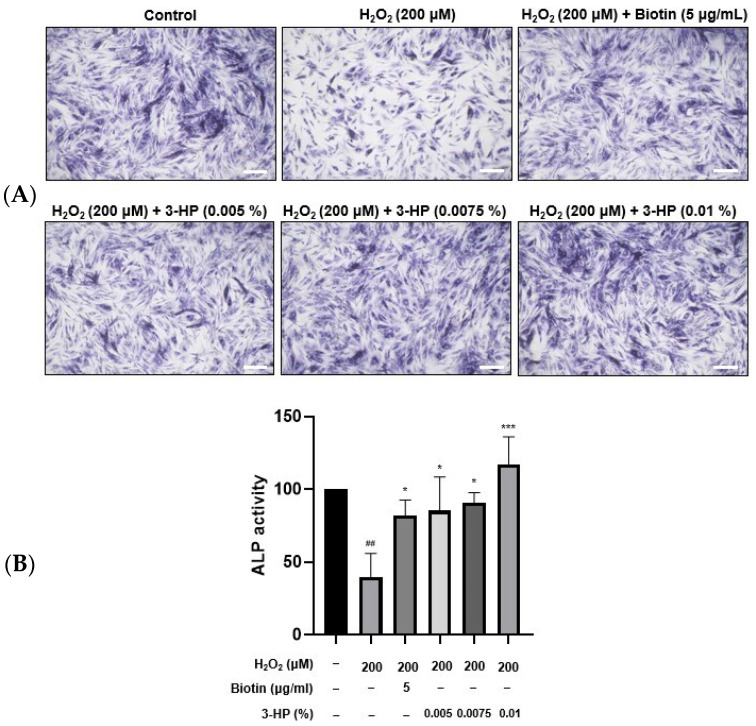
The effects of 3-HP on the ALP activity in H_2_O_2_-damaged HFDPCs. (**A**) HFDPCs exposed to oxidative stress by H_2_O_2_ were pretreated with 5 μg/mL biotin or 0.005-0.01% 3-HP, then incubated with ALP staining solution for 18 h. (**B**) The quantitative analysis of ALP activity in each group was shown. Statistical significance was determined using one-way ANOVA followed by Tukey’s post hoc test. Data were representative of three independent experiments. ## *p* < 0.01 vs. control group, * *p* < 0.05, *** *p* < 0.001 vs. H_2_O_2_-treated group. Scale bar: 20 µm.

ALP plays an important role in stimulating hair follicle development and initiating hair shaft formation [35,36]. Its enzymatic activity is regarded as a reliable biomarker of the hair-inductive potential of HFDPCs, as higher ALP expression typically reflects enhanced folliculogenic capability [37]. This is particularly evident during the anagen phase, where DPCs exhibit elevated ALP levels in parallel with increased proliferation and differentiation cues within the follicular microenvironment [38]. HFDPCs play a crucial role in transitioning hair follicles from the catagen phase to the anagen phase. 5 μg/mL biotin treatment significantly enhanced ALP expression compared with the H_2_O_2_-damaged HFDPC group. Similarly, treatment with 0.01% 3-HP also led to a notable upregulation of ALP expression (Figure 3A,B).

### 2.4. 3-HP Reduced ROS Levels in H_2_O_2_-Damaged HFDPCs

**Figure 4:**
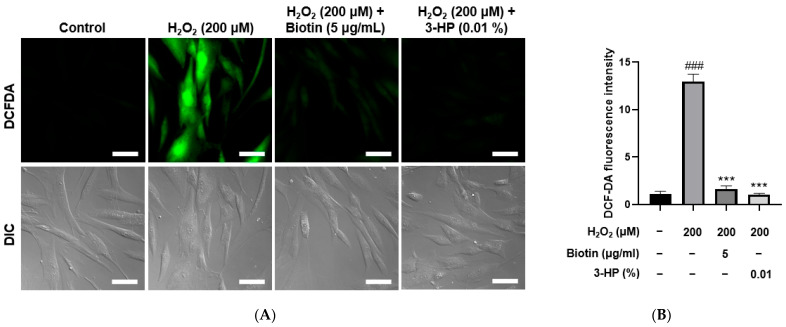
The effect of 3-HP on intracellular ROS reduction in H_2_O_2_-damaged HFDPCs. (**A**) Representative fluorescence images of intracellular ROS in HFDPCs treated with 200 μM H_2_O_2_, followed by pretreatment with 5 μg/mL biotin, 100 μg/mL, and 0.01% 3-HP. ROS levels were visualized as green using the fluorescent probe DCF-DA assay. (**B**) The quantitative analysis of fluorescence intensity in each group was shown. Data were presented as mean ± SD from three independent experiments. ### *p* < 0.001 vs. control group; *** *p* < 0.001 vs. H_2_O_2_-treated group. Scale bar: 50 μm.

Excessive accumulation of ROS is known to induce oxidative stress, which can impair the functional capacity of dermal papilla cells to support and promote hair growth [39]. Using the DCF-DA assay, intracellular ROS generation was stimulated by treatment with 200 μM H_2_O_2_. As expected, the ROS levels in the positive control groups treated with 5 μg/mL biotin were significantly reduced compared with the H_2_O_2_-treated group. Similarly, cells treated with 0.01% 3-HP also exhibited a marked decrease in ROS levels (Figure 4A,B). Fluorescence quantification showed that 3-HP markedly reduced DCF-DA-derived fluorescence compared with the H_2_O_2_-treated group, meaning that 3-HP effectively diminished intracellular ROS accumulation in oxidatively stressed HFDPCs. These results suggest that 3-HP exerted a protective effect by attenuating H_2_O_2_-induced oxidative burden within the cells.

### 2.5. 3-HP Activated the Nrf2 Pathway in H_2_O_2_-Damaged HFDPCs

**Figure 5:**
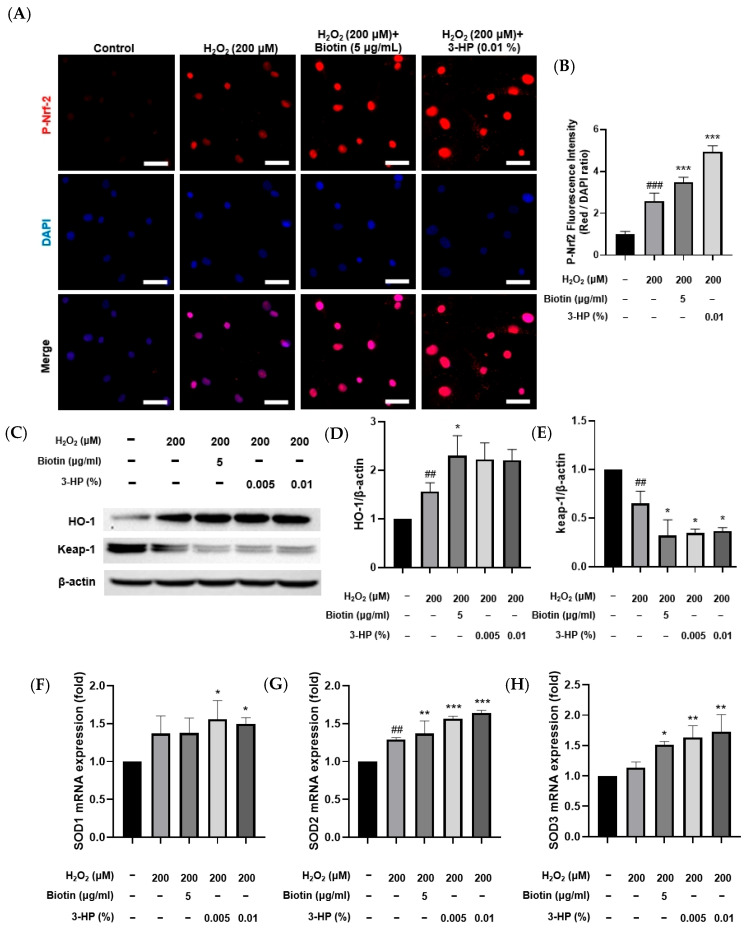
The effect of 3-HP on Nrf2 activation and antioxidant defense regulation in H_2_O_2_-damaged HFDPCs. (**A**) Representative mmunofluorescence images showing p-Nrf2 localization (red) in HFDPCs treated with H_2_O_2_ with or without biotin or 3-HP; nuclei were counterstained with DAPI (blue). (**B**) Quantification of p-Nrf2/DAPI fluorescence intensity. (**C**–**E**) Western blot and densitometric analysis of HO-1 and Keap-1 protein levels following H_2_O_2_ and 3-HP treatment. (**F**–**H**) Relative mRNA levels of SOD1, SOD2, and SOD3 measured by qRT-PCR under oxidative stress conditions. Data were expressed as mean ± SD from three independent experiments. ## *p* < 0.01, ### *p* < 0.001 vs. control group; * *p* < 0.05, ** *p* < 0.01, *** *p* < 0.001 vs. H_2_O_2_-treated group. Scale bar: 50 μm.

In response to elevated oxidative stress, Nrf2 activation initiates the transcriptional upregulation of antioxidant enzymes, which in turn mitigate oxidative stress–induced cellular injury [40,41,42]. Consistent with this mechanism, treatment with 3-HP markedly increased p-Nrf2 levels compared with the H_2_O_2_-treated group, indicating that 3-HP enhanced the cellular antioxidant response by promoting Nrf2 signaling (Figure 5A,B). To further investigate antioxidant regulation at the protein level, HO-1 and Keap-1 expression were first analyzed by Western blot analysis. HO-1 protein abundance increased following H_2_O_2_ exposure relative to the control group, reflecting an adaptive cytoprotective response to oxidative stress. Pretreatment with 3-HP led to a more pronounced elevation in HO-1 expression, indicating that 3-HP strengthened the Nrf2-mediated antioxidant response during oxidative stress (Figure 5C,D). In contrast, Keap-1 showed the highest abundance under basal conditions, reflecting its role as the primary suppressor that maintained Nrf2 in an inactive state during homeostasis [43]. Exposure to H_2_O_2_ reduced Keap-1 protein levels, likely through oxidative modification of its cysteine residues and subsequent protein destabilization [44]. This decline became even more pronounced when cells were pretreated with 3-HP, suggesting that 3-HP further destabilized Keap-1, thereby enabling more sustained Nrf2 activation under oxidative stress conditions (Figure 5E). To assess transcriptional regulation, mRNA expression of SOD isoforms was measured. Cells pretreated with 3-HP showed significant upregulation of all SOD isoforms, indicating that 3-HP strengthens the antioxidant defense through enhanced mitochondrial ROS clearance and Nrf2-dependent transcription (Figure 5F–H). These data demonstrated that 3-HP reinforces multiple levels of the Nrf2 antioxidant defense system by promoting Nrf2 phosphorylation, upregulating antioxidant enzymes, enhancing HO-1 induction, and reducing Keap-1 expression, thereby mitigating oxidative stress-induced dysfunction in HFDPCs.

### 2.6. 3-HP Enhanced Mitochondrial Function in H_2_O_2_-Damaged HFDPCs

Mitochondrial activity is essential for controlling hair follicle development [45]. It is well established that the activation of mitochondrial respiration and ATP production contributes to the promotion of hair growth [46,47]. The mitochondrial membrane potential serves as an important measure of both mitochondrial performance and overall cellular health. JC-1 staining is a widely utilized method to assess ΔΨm, where healthy mitochondria with intact membrane potential show red fluorescence (JC-1 aggregates), whereas depolarized mitochondria exhibit green fluorescence (JC-1 monomers) [48]. Thus, the red/green fluorescence ratio serves as a reliable measure of mitochondrial integrity and bioenergetic status [49]. Intracellular ATP levels are a direct measure of mitochondrial bioenergetic function and overall cellular viability [50,51]. A reduction in ATP production is indicative of mitochondrial impairment, while maintenance of ATP levels reflects preserved mitochondrial function [52].

**Figure 6:**
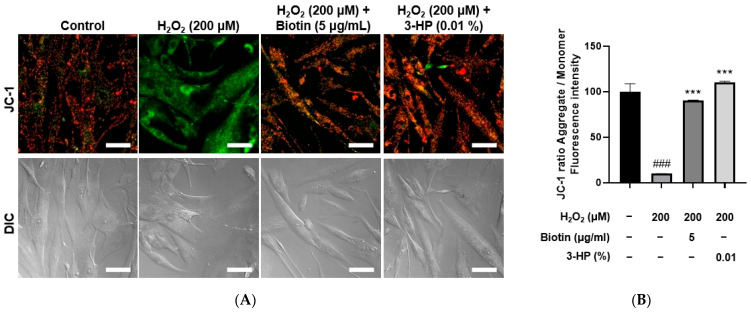
The influences of 3-HP on mitochondrial membrane potential in H_2_O_2_-damaged HFDPCs. (**A**) Representative JC-1 fluorescence images showing mitochondrial membrane potential in HFDPCs treated with H_2_O_2_ alone, or pretreated with 5 μg/mL biotin, 100 μg/mL, and 0.01% 3-HP. High mitochondrial membrane potential is represented by red fluorescence, whereas depolarized mitochondria are visualized as green fluorescence. (**B**) The quantitative analysis of red/green fluorescence ratios in each group was shown. Data were presented as mean ± SD from three independent experiments. ### *p* < 0.001 vs. control group; *** *p* < 0.001 vs. H_2_O_2_-treated group. Scale bar: 50 μm.

To evaluate the effects of 3-HP on mitochondrial function under oxidative stress conditions, HFDPCs were treated with 200 μM H_2_O_2_, and mitochondrial membrane potential and ATP production were assessed using JC-1 and Live Cell ATP assays, respectively. H_2_O_2_ treatment significantly disrupted mitochondrial membrane potential, as indicated by a decreased red-to-green fluorescence ratio in the JC-1 assay. However, pretreatment with 0.01% 3-HP markedly restored the membrane potential, comparable to the effects observed with 5 μg/mL biotin (Figure 6A,B). Similarly, ATP levels were significantly reduced in the H_2_O_2_-treated group, whereas both the 3-HP and positive control groups showed a substantial recovery in intracellular ATP production (Supplementary Figure S1). These results indicated that 3-HP alleviated H_2_O_2_-induced mitochondrial dysfunction and supported mitochondrial bioenergetics in HFDPCs. To further substantiate the restorative effects of 3-HP on mitochondrial activity, mitochondrial ROS production was quantified using MitoSOX Red fluorescence. H_2_O_2_ exposure led to a pronounced elevation in mitochondrial superoxide levels, confirming oxidative injury within the organelle. However, pre-treated cells with 0.01% 3-HP exhibited a significant reduction in MitoSOX fluorescence intensity, comparable to that observed in the biotin group (Supplementary Figure S2). These data demonstrated that 3-HP not only stabilized mitochondrial membrane potential and preserved ATP synthesis, but also effectively suppressed excessive mitochondrial ROS generation.

### 2.7. 3-HP Enhanced Hair Growth-Related Signaling Pathways in H_2_O_2_-Damaged HFDPCs

As a major element of the MAPK cascade, the extracellular signal-regulated kinase (ERK) pathway is essential for regulating cellular growth, differentiation, and viability [53,54]. Activation of this pathway is closely associated with the promotion of the anagen phase and the overall regeneration of hair follicles [55,56]. Glycogen synthase kinase 3 beta (GSK-3β) is a serine/threonine kinase involved in various cellular signaling pathways, including the Wnt/β-catenin pathway [57,58]. By inhibiting β-catenin breakdown, GSK-3β facilitates its cytoplasmic build-up and nuclear entry, thereby triggering Wnt target genes involved in cellular proliferation and survival [59,60]. As a key downstream component of Wnt signaling, β-catenin, upon nuclear accumulation, partners with TCF/LEF factors to modulate gene expression related to cellular growth and tissue maintenance [61,62]. In the context of hair biology, nuclear β-catenin accumulation is critical for hair follicle morphogenesis and the maintenance of the anagen phase [63,64]. These pathways are integral to hair growth and anagen phase maintenance.

**Figure 7:**
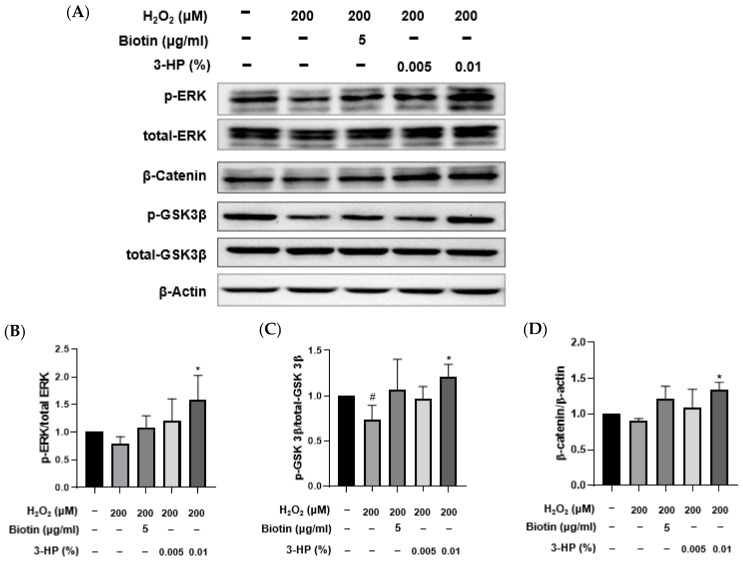
The effect of 3-HP on the expression of hair growth-related signaling proteins in H_2_O_2_-damaged HFDPCs. (**A**) Representative images of HFDPCs showing the expression of phosphorylated ERK (p-ERK), total ERK, phosphorylated GSK3β (p-GSK3β), total GSK3β, β-catenin, and β-actin. Cells were pretreated with 5 μg/mL biotin or 0.01% 3-HP, followed by exposure to 200 μM H_2_O_2_. (**B**–**D**) The quantitative analysis of each group was shown. Data were presented as mean ± SD from three independent experiments. # *p* < 0.05 vs. control group; * *p* < 0.05 vs. H_2_O_2_-treated group.

To investigate the molecular mechanisms by which 3-HP exerts protective and pro-regenerative effects in HFDPCs under oxidative stress, the activation of ERK and GSK3β signaling, as well as β-catenin accumulation, was assessed by Western blot analysis (Figure 6A). Consistent with oxidative stress effects, H_2_O_2_ exposure led to a significant decrease in ERK and GSK3β phosphorylation and reduced β-catenin levels relative to control. Pretreatment with 3-HP, however, restored phosphorylation of ERK and GSK3β (Figure 7B,C) and markedly elevated β-catenin expression compared with the H_2_O_2_ group, suggesting enhanced activation of Wnt/β-catenin signaling (Figure 7D). These effects were comparable to those observed with biotin treatment, a known hair growth-promoting agent. These findings indicated that 3-HP modulated key signaling pathways involved in hair follicle regeneration in oxidative stress-damaged HFDPCs.

### 2.8. 3-HP Enhanced the Expression of Hair Growth-Related Genes in H_2_O_2_-Damaged HFDPCs

DKK-1 negatively regulates the Wnt/β-catenin pathway, promoting hair follicle regression from the anagen phase to the catagen phase, which limits hair proliferation [65] In contrast, insulin-like growth factor 1 (IGF-1) and WNT10B act as positive regulators of hair follicle development by enhancing follicular proliferation and promoting the telogen-to-anagen transition, respectively, with IGF-1 prolonging the anagen phase and WNT10B activating the canonical Wnt/β-catenin signaling pathway [66,67]. DKK-1 is a known antagonist of the Wnt/β-catenin signaling pathway [68]. Elevated levels of DKK-1 have been implicated in the regression of hair follicles and the promotion of the catagen phase, leading to hair loss [69,70]. IGF-1 is a growth factor that promotes cell proliferation and survival [71]. In the context of hair biology, IGF-1 has been shown to stimulate the proliferation of dermal papilla cells and promote hair follicle development [72]. WNT10B is a member of the Wnt family of proteins and plays a crucial role in activating the Wnt/β-catenin signaling pathway, which is essential for hair follicle development and regeneration [73]. Under oxidative stress conditions in HFDPCs, altering the expression of these genes may substantially impact hair follicle regeneration. To examine the transcriptional levels of 3-HP on oxidative stress-induced damage in HFDPCs, we examined the mRNA expression levels of key hair growth-related genes, including DKK-1, IGF-1, and WNT10B, using quantitative real-time PCR.

**Figure 8:**
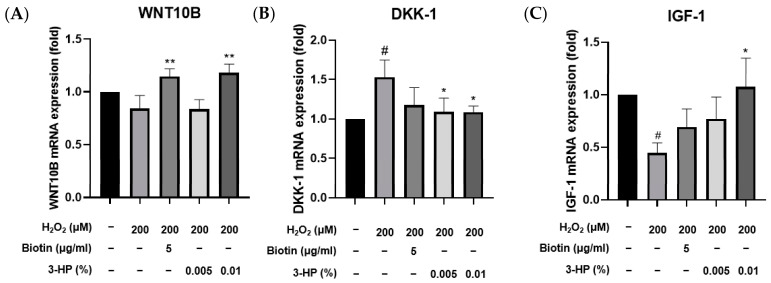
Effects of 3-HP on mRNA expression of hair growth genes in H_2_O_2_-challenged HFDPCs. Pretreatment was performed with 5 μg/mL biotin or 0.01% 3-HP for 24 h, followed by 2 h of 200 μM H_2_O_2_. Gene expression of (**A**) DKK-1, (**B**) IGF-1, and (**C**) WNT10B was assessed using qRT-PCR. The results were normalized to β-actin expression and are presented as fold changes relative to the H_2_O_2_-treated group. Data were expressed as mean ± SD from three independent experiments. # *p* < 0.05 vs. control group; * *p* < 0.05, ** *p* < 0.01, compared with H_2_O_2_-treated group.

Treatment with 3-HP notably suppressed the expression of DKK-1, a gene commonly upregulated in response to oxidative damage (Figure 8B). Conversely, the mRNA expression levels of IGF-1 and WNT10B, which are known to promote hair follicle development and anagen phase induction [74], were markedly increased following treatment with 0.01% 3-HP (Figure 8A,C). These findings indicate that 3-HP effectively regulates the expression of genes associated with follicular regeneration and may counteract the inhibitory effects of oxidative stress on hair growth.

### 2.9. 3-HP Enhanced the Size of 3D Spheroids Formed by H_2_O_2_-Treated HFDPCs

Spheroid culture of dermal papilla cells has been reported to better preserve their gene expression patterns and hair-inductive capabilities, offering a more physiologically relevant in vitro model for evaluating dermal papilla function [75].

**Figure 9:**
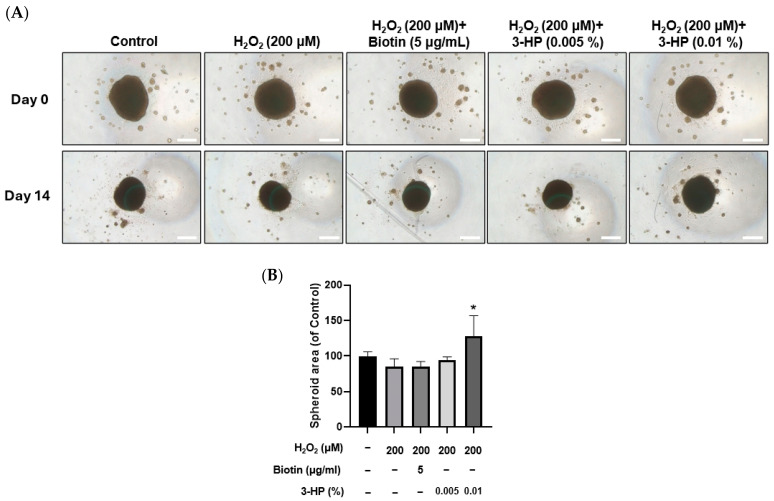
Effects of 3-HP on 3D spheroid formation in H_2_O_2_-damaged HFDPCs. (**A**) Representative images of HFDPCs spheroids at Day 0 and Day 14 following treatment with 200 µM H_2_O_2_, with or without pretreatment with 5 µg/mL biotin or 3-HP (0.005% or 0.01%). Scale bar = 200 µm. (**B**) The quantitative analysis of the spheroid area was shown. Data were expressed as mean ± SD (n = 3). * *p* < 0.05 vs. H_2_O_2_-treated group.

In this system, reductions in spheroid size are closely linked to impaired cell viability, indicating that spheroid diameter or area is a meaningful indicator of functional status [76]. In H_2_O_2_-treated HFDPCs, pretreatment with 3-HP markedly increased the diameter and area of 3D spheroids compared with the H_2_O_2_-treated group (Figure 9A,B). Since three-dimensional spheroids of dermal papilla cells retain essential hair-inductive gene expression and even possess the ability to trigger follicle neogenesis [77,78], the enlargement of spheroids by 3-HP indicates a functional restoration of DP inductivity under oxidative stress conditions.

## 3. Discussion

Previous research has highlighted the essential role of HFDPCs in initiating and maintaining the anagen phase of the hair cycle through the secretion of growth factors and extracellular matrix components [79,80].

In this study, we demonstrated that 3-HP effectively promoted hair growth-related functions in HFDPCs, particularly under oxidative stress conditions induced by H_2_O_2_. These findings provide valuable insights into the potential application of 3-HP as a therapeutic agent for hair loss treatment. Importantly, we observed that 3-HP significantly increased the expression of ALP, a well-established marker of dermal papilla cell inductivity (Figure 3). Moreover, 3-HP reduced intracellular ROS accumulation, which is known to impair the function and viability of HFDPCs, thereby protecting the cells from H_2_O_2_-induced oxidative damage. In parallel, 3-HP treatment enhanced the nuclear translocation of Nrf2, indicating that its protective effect is mediated through activation of the Nrf2-mediated antioxidant pathway (Figure 4). Mitochondrial integrity plays a critical role in maintaining the bioenergetic capacity of HFDPCs [81]. Mitochondrial membrane potential was evaluated using the JC-1 assay. In polarized, healthy mitochondria, JC-1 accumulates and forms aggregates that emit red fluorescence, whereas in depolarized mitochondria with reduced membrane potential, JC-1 remains monomeric and emits green fluorescence [82]. The ratio of red to green fluorescence thus serves as a reliable measure of mitochondrial integrity. Furthermore, the intracellular ATP level was measured using the ATP assay to evaluate the bioenergetic function of mitochondria [83].

This suggests that 3-HP may exert its protective effects by sustaining mitochondrial function, which in turn supports cellular activities essential for hair follicle regeneration (Figure 6). Western blot analysis further revealed that 3-HP induced the activation of ERK and GSK3β/β-catenin signaling pathways, both of which are pivotal in regulating proliferation and differentiation in HFDPCs (Figure 7). While the AKT pathway is recognized for its role in supporting cell survival and hair follicle inductivity, no significant changes in AKT activation, assessed via p-AKT/AKT levels, were detected under our experimental conditions. This discrepancy warrants further investigation to elucidate whether 3-HP modulates AKT signaling under different temporal or dosage parameters. The ERK signaling pathway is essential for regulating survival, cell proliferation, and migration, processes that are critical for initiating and maintaining the anagen phase of the hair cycle [84,85]. Activation of ERK signaling in HFDPCs has been shown to enhance the secretion of key growth factors such as VEGF and IGF-1, thereby stimulating hair shaft elongation [86].

Meanwhile, GSK3β/β-catenin signaling is crucial for the development and regenerative processes of hair follicles [87]. Inhibiting GSK3β through phosphorylation allows β-catenin to build up in the cytoplasm and move into the nucleus, where it stimulates Wnt target genes to support HFDPC inductivity, follicular growth, and stem cell activation [88,89]. Therefore, the upregulation of these pathways by 3-HP means a strong potential to enhance the biological functionality of HFDPCs and promote hair regeneration. 3-HP treatment increased p-GSK3β and nuclear β-catenin expression, suggesting an association with Wnt/β-catenin pathway activation. However, direct activation remains to be verified using pathway inhibitors or reporter assays in future studies. Additionally, 3-HP treatment enhanced IGF-1 and WNT10B expression while reducing levels of DKK-1, a negative regulator of Wnt signaling (Figure 8). These findings indicate that 3-HP not only protects HFDPCs from oxidative stress but also promotes a gene expression profile conducive to hair regeneration [90]. Under oxidative stress conditions, 3-HP treatment resulted in enlarged HFDPC spheroids (Figure 9), indicating a partial restoration of dermal papilla inductive potential. Although spheroid size alone cannot fully confirm folliculogenic capacity, these results indicate that 3-HP may help maintain the 3D organization and functional activity of dermal papilla cells essential for hair inductivity [91,92].

**Figure 10:**
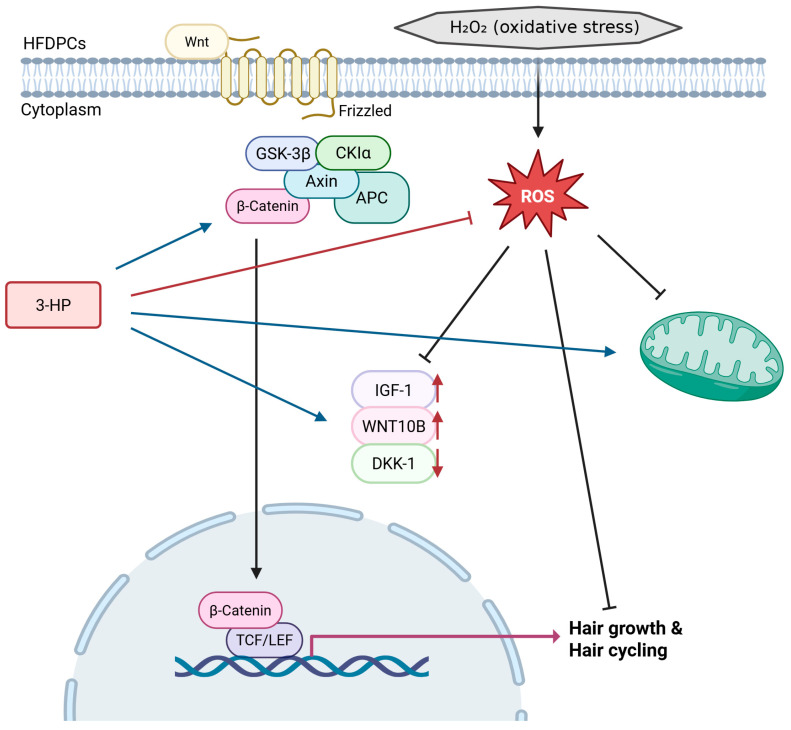
Schematic representation of the proposed mechanism through which 3-HP alleviates H_2_O_2_-induced oxidative stress and stimulates Wnt/β-catenin signaling to enhance hair growth and regulate hair cycling. (created with BioRender, https://www.biorender.com/ accessed on 31 December 2025).

Taken together, our results emphasize the regenerative relevance of 3-HP in promoting hair follicle regeneration through modulation of the dermal papilla microenvironment (Figure 10). This activity supports its potential application as an active ingredient in topical formulations, cosmeceuticals, and pharmacological strategies for alopecia management. However, further studies using in vivo models and well-controlled clinical trials are essential to clarify the clinical relevance and safety profile of 3-HP. Such investigations will help confirm its efficacy, optimize its formulation, and potentially pave the way for its translation into dermatological therapeutics for treating various forms of hair loss, including stress-induced alopecia [93,94].

## 4. Materials and Methods

### 4.1. Chemicals and Reagents

The 3-HP was supplied by LG Chem (Seoul, Republic of Korea). It was produced via microbial fermentation using a genetically engineered strain of Escherichia coli, with glycerol as the carbon source. 3-HP was produced in salt form via fermentation at neutral pH. Biomass was removed by microfiltration, and the 3-HP salt was recovered through crystallization. It was then converted into its acid form via acidification. Finally, 3-HP was concentrated and recovered as a 70% aqueous solution with a purity of 99.5%. Biotin at a concentration of 5 μg/mL served as a positive control because it is a well-established micronutrient known to support hair follicle function [95,96]. Biotin supports keratin structure and has been widely applied as a reference micronutrient in in vitro studies assessing dermal papilla cell function and hair-growth-related responses [97,98].

### 4.2. Cell Culture

HFDPCs sourced from PromoCell (Heidelberg, Germany) were maintained in follicle dermal papilla cell growth medium containing a supplement mix and 1% penicillin–streptomycin at 37 °C in a humidified 5% CO_2_ atmosphere. Ready-to-use HFDPC Medium and a Detach Kit from the same supplier were used for cell handling. The Detach Kit included Trypsin/EDTA Solution, Trypsin Neutralization Solution, and HEPES BSS Solution.

### 4.3. Cell Viability Assay

HFDPC viability was measured using the EZ-CytoX assay (DoGenBio, Seoul, Republic of Korea), with cells seeded at 2 × 10^4^ cells per well in 96-well plates and treated with various concentrations of 3-hydroxypropionic acid (3-HP; 0.001–0.01%) for 24 h. Following treatment, EZ-Cytox solution was added to each well and incubated for 2 h at 37 °C. Absorbance was measured using a BioTek Multi-Mode Microplate Reader (Winooski, VT, USA), and cell viability was calculated as described below:

Cell viability (%) = (OD_treated_/OD_control_) × 100

where ${\mathrm{OD}}_{\mathrm{treated}}$ and ${\mathrm{OD}}_{\mathrm{control}}$ represent the optical density values of the treated and untreated (control) groups, respectively.

### 4.4. Wound Healing Assay

Confluent HFDPC monolayers (>90%) in 6-well plates were scratched at the center of each well using a 200 µL pipette tip. The time of scratching was set as 0 h. The cells were treated with 200 µM H_2_O_2_, 5 µg/mL biotin, and 0.005–0.01% 3-HP. Phase-contrast images were captured after 24 h using an ECLIPSE Ts2 microscope (Nikon, Tokyo, Japan) for both treated and control groups.

### 4.5. Alkaline Phosphatase Staining (ALP) Assay

Alkaline Phosphatase Staining was assessed by an Alkaline Phosphatase Staining Kit (Abcam, Cambridge, UK). HFDPCs were seeded in a 24-well plate. HFDPCs were treated with 200 µM H_2_O_2_, 5 µg/mL biotin, and 0.01% 3-HP. A total of 0.4 mL of fixing solution was added to each well, followed by a 5 min incubation. The wells were then washed twice with 1× PBST. After AP staining (0.4 mL per well, 6 h, dark), images were captured using an ECLIPSE Ts2 microscope (Nikon, Tokyo, Japan) and analyzed with Fiji ImageJ software, version 1.53e (Windows 64-bit; NIH, Bethesda, MD, USA).

### 4.6. Measurement of Reactive Oxygen Species (ROS)

ROS generation within HFDPCs was assessed with an H2DCFDA-based fluorescence assay (Abcam, Cambridge, UK). Cells seeded on confocal dishes were cultured for 24 h and subsequently treated with 200 µM H_2_O_2_ in the presence or absence of 5 µg/mL biotin or 0.01% 3-HP. After DCF-DA loading (10 µM, 15 min, dark), fluorescence images were acquired with a Nikon Eclipse Ti2 microscope (Nikon, Tokyo, Japan).

### 4.7. Immunofluorescence Analysis

HFDPCs were plated in confocal dishes at a density of 5.0 × 10^4^ cells per well and cultured. The cells were subsequently exposed to 5 µg/mL biotin, 0.01% 3-HP. Immunofluorescence staining was performed following fixation, permeabilization, and blocking, with p-Nrf2 detected using a primary antibody at 4 °C overnight and an HRP-conjugated secondary antibody at 37 °C. Nuclei were counterstained with DAPI, and images were acquired using a Nikon Eclipse Ti2 microscope (Nikon, Tokyo, Japan).

### 4.8. Measurement of Mitochondrial Membrane Potential (JC-1)

Mitochondrial membrane potential in HFDPCs was analyzed with the JC-1 Assay Kit (Abcam, Cambridge, UK). Cells seeded on confocal dishes were cultured for 24 h before treatment with 200 µM H_2_O_2_, 5 µg/mL biotin, or 0.01% 3-HP. Cells were washed with fresh culture medium and stained with 10 µM JC-1 dye. The cells were then incubated for 20 min, protected from light at 37 °C. Changes in mitochondrial membrane potential were quantified as the red-to-green fluorescence ratio of JC-1 aggregates and monomers, respectively, acquired with a Nikon Eclipse Ti2 fluorescence microscope (Nikon, Tokyo, Japan).

### 4.9. Measurement of Mitochondrial ATP Content and Mitochondrial ROS

Cellular ATP levels and mitochondrial ROS in live HFDPCs were independently assessed using a luminescence-based Cell Meter^TM^ ATP assay (AAT Bioquest, Pleasanton, CA, USA), and the MitoSOX^TM^ Red mitochondrial superoxide indicator (Thermo Fisher Scientific, Waltham, MA, USA), respectively. In each experiment, MitoLite^TM^ Green FM (AAT Bioquest, Pleasanton, CA, USA) was applied concurrently to visualize mitochondria and confirm cell viability and localization. Cells seeded in confocal dishes were maintained for 24 h before measurement. Following treatment with 200 µM H_2_O_2_, 5 µg/mL biotin, and 0.01% 3-HP, the culture medium was removed and replaced with the ATP assay working solution or the MitoSOX^TM^ staining solution, each containing MitoLite^TM^ Green FM, according to the manufacturer’s instructions. After incubation at 37 °C for 30 min in the dark, the cells were washed with DPBS. Fluorescence images were obtained using a Nikon Eclipse Ti2 microscope (Nikon, Tokyo, Japan).

### 4.10. Western Blot Analysis

HFDPCs were incubated with the indicated samples for 24 h and subsequently challenged with 200 μM H_2_O_2_ for 2 h. Cells were rinsed with PBS and lysed in RIPA buffer, and total protein content was quantified using a BCA assay kit (Thermo Fisher Scientific, Waltham, MA, USA). Equivalent protein amounts (20 μg) were resolved by SDS–PAGE and electrotransferred onto PVDF membranes. Non-specific binding was suppressed by blocking membranes with 5% non-fat milk in TBS-T for 2 h at room temperature. Primary antibodies against p-ERK, ERK, and β-actin (Cell Signaling Technology, Danvers, MA, USA), as well as β-catenin, p-GSK3β, and GSK3β (Santa Cruz Biotechnology, Carlsbad, CA, USA), were applied overnight at 4 °C. Membranes were then incubated with HRP-linked secondary antibodies for 2 h at room temperature. Western blot experiments were independently repeated three times. Protein bands were visualized using an enhanced chemiluminescence detection system and imaged with the iBright 1500 Imaging System (Thermo Fisher Scientific, Waltham, MA, USA). Band intensities were quantified using Fiji ImageJ software (version 1.53e).

### 4.11. Real-Time Quantitative Reverse Transcriptase-Polymerase Chain Reaction

HFDPCs prepared for qRT-PCR analysis were allowed to attach in 6-well plates (5 × 10^4^ cells/mL) for 24 h, after which oxidative stress was induced using 200 µM H_2_O_2_ in the presence or absence of 5 µg/mL biotin or 0.01% 3-HP for an additional 24 h.

Total RNA was then isolated using TRIzol reagent (Thermo Fisher Scientific, Waltham, MA, USA), according to the manufacturer’s instructions. Total RNA (2 µg) was reverse-transcribed to cDNA using a commercial synthesis kit (Thermo Fisher Scientific, Waltham, MA, USA). Gene expression was subsequently analyzed by qRT-PCR employing TaqMan Universal Master Mix II with UNG. The PCR mixture was prepared by combining synthesized cDNA, DEPC-treated water, TaqMan Universal Master Mix II, and gene-specific TaqMan primers at the indicated volumes.

### 4.12. Statistical Analyses

Results obtained from at least three independent experiments were summarized as mean ± SD. Group comparisons were conducted using one-way ANOVA with Tukey’s post hoc correction implemented in GraphPad Prism (version 8.0.1). Assumptions of normality and equal variance were examined before analysis, and *p*-values below 0.05 were considered significant.

## 5. Conclusions

This study demonstrated that 3-HP effectively protected human follicle dermal papilla cells from oxidative stress–induced dysfunction. By restoring mitochondrial activity, reducing intracellular oxidative burden, and activating hair growth–related signaling pathways, 3-HP supported the maintenance of dermal papilla cell inductivity under stress conditions. These findings suggest that 3-HP holds promise as a functional cosmetic ingredient for managing oxidative stress–associated hair thinning.

## Data Availability

The data from this study are available upon demand from the corresponding author.

## Supplementary Materials

The following supporting information can be downloaded at: https://www.mdpi.com/article/10.3390/ijms27031480/s1.

## Abbreviations

HFDPCs	Human Follicle Dermal Papilla Cells
H_2_O_2_	Hydrogen peroxide
DCFDA	2′,7′-dichlorodihydrofluorescein diacetate
ROS	reactive oxygen species
FITC	Fluorescein isothiocyanate
RITC	Rhodamine B isothiocyanate
JC-1	5,5′,6,6′-tetrachloro-1,1′,3,3′-tetraethylbenzimidazolocarbo-cyanine iodide
IF	immunofluorescence
BCA	bicinchoninic acid
ERK	extracellular signal-regulated kinase
Nrf2	nuclear factor erythroid 2-related factor 2
GSK3β	Glycogen synthase kinase-3 beta
IGF-1	Insulin-like growth factor 1
DKK-1	Dickkopf-1
3-HP	3-hydroxypropionic acid
