# Supplementary material for: 3-Hydroxypropionic Acid Enhances Hair Growth-Related Signaling in Human Follicle Dermal Papilla Cells via Activation of the Wnt/β-Catenin Pathway
Source: Int J Mol Sci. 2026 Feb 2;27(3):1480. doi: 10.3390/ijms27031480 (PMC12898851; doi:10.3390/ijms27031480)
Supplement: Supplementary file 1 [file ijms-27-01480-s001.zip › ijms-3876940-supplementary.pdf]

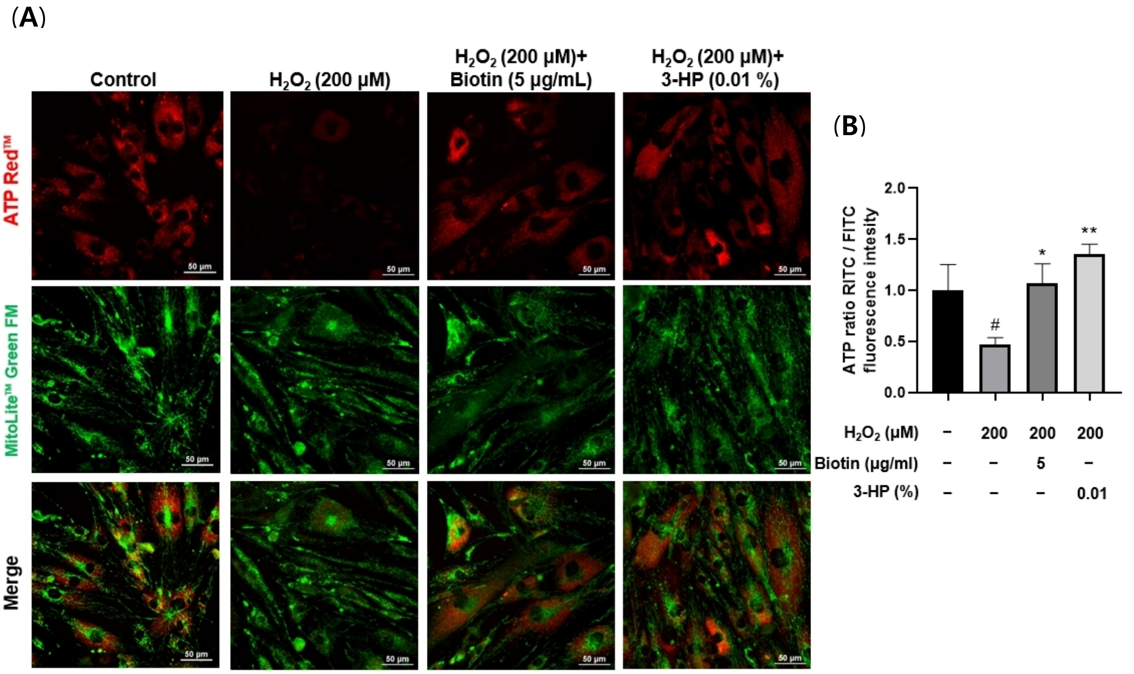

Supplementary Figure S1. The effect of 3-HP on restoring ATP levels in H_2O_2-damaged HFDPCs.

(A) Representative fluorescence images from the Live Cell ATP assay showing intracellular ATP levels under the same treatment conditions. Red fluorescence represents ATP levels, while green fluorescence indicates mitochondria. Images represent one of three independent experiments. (B) ATP levels were analyzed using Image J software, version 1.53e. Quantitative analysis of ATP fluorescence intensity was shown. Data mean ± SD from three independent experiments. #$p < 0.05$ vs. control group; *$p < 0.05$, **$p < 0.01$ vs. H_2O_2-treated group. Scale bar: 50 μm.

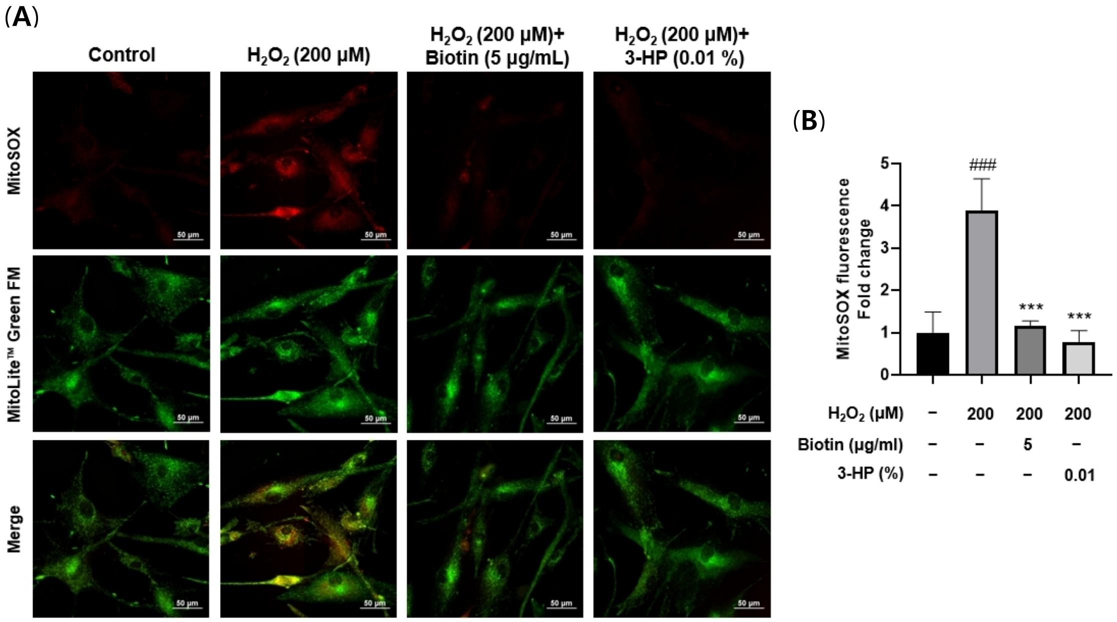

Supplementary Figure S 2. The inhibitory effect of 3-HP on mitochondrial ROS production in H_2O_2-damaged HFDPCs.

(A) Representative fluorescence images from the MitoSOX assay under the same treatment conditions. Red fluorescence represents mitochondrial ROS levels, while green fluorescence indicates mitochondria. Images represent one of three independent experiments. (B) Mitochondrial ROS levels were analyzed using Image J software, version 1.53e. Quantitative analysis of mitochondrial fluorescence intensity was shown. Data mean ± SD from three independent experiments. ###$p < 0.001$ vs. control group; ***$p < 0.001$ vs. H_2O_2-treated group. Scale bar: 50 μm.
